# Hospital at Home for older residents with physical deterioration: care home staff experiences

**DOI:** 10.1093/ageing/afag236

**Published:** 2026-08-11

**Authors:** Chidiebere Nwolise, Sara McKelvie, Siabhainn Russell, Michele Peters

**Affiliations:** Nuffield Department of Population Health, University of Oxford, Richard Doll Building, Old Road Campus, Oxford, Oxfordshire OX3 7LF, UK; Primary Care Research Centre, University of Southampton, Southampton SO17 1BJ, UK; Nuffield Department of Population Health, University of Oxford, Richard Doll Building, Old Road Campus, Oxford, Oxfordshire OX3 7LF, UK; Nuffield Department of Population Health, University of Oxford, Richard Doll Building, Old Road Campus, Oxford, Oxfordshire OX3 7LF, UK

**Keywords:** Hospital at Home, care homes, older adults, qualitative research, service delivery

## Abstract

**Introduction:**

Health and social care systems face rising pressures due to population ageing, rising frailty, multimorbidity and complex care needs. Traditional hospital-based care is increasingly recognised as unsustainable, and may be inappropriate for older adults living in care homes. In England, policy promotes shifting care from acute hospitals to community and residential settings. Hospital at Home (HaH) delivers hospital-level care in a person’s usual residence; however, little is known about its delivery and experience within care homes.

**Methods:**

This study reports qualitative findings from semi-structured interviews with care home staff, supported by descriptive survey data and open-ended survey responses. Care home staff were recruited from several localities in the South of England. Quantitative survey data were analysed descriptively and qualitative data, thematically.

**Results:**

Fifty participants completed the survey and 20 participated in interviews. Hospital admission remained necessary for some residents, particularly for fractures and acute emergencies. However, hospitalisation was frequently associated with distress, communication breakdowns and hospital-associated decline. In contrast, HaH was perceived to offer benefits including collaborative working, improved continuity, preservation of dignity and comfort and reduced exposure to hospital-related risks. Key barriers included workforce capacity, geographical coverage, limited opening hours and inconsistent referral pathways, restricting timely and equitable access.

**Conclusion:**

Hospital at Home was perceived by care home staff as a valuable and often preferable alternative to hospitalisation for many residents. However, structural and organisational barriers must be addressed to realise its full potential within care home settings. Future research should examine how HaH can be more widely implemented in care homes.

## Key Points

- Hospital at Home (HaH) is perceived by care home staff to reduce distress and hospital-associated decline while supporting dignity, comfort and continuity of care for care home residents.
- Care home staff strongly value HaH but report significant limitations in access and service capacity.
- Wider implementation of HaH requires investment in workforce, infrastructure and training to support sustainable delivery in care homes.

## Introduction

Health and social care systems are under increasing strain as the population ages and levels of frailty, multimorbidity and complex care needs rise, placing growing demand on acute hospital services [1–3]. Traditional models of hospital-based care are increasingly recognised as unsustainable and, in some cases, inappropriate for older people who may be more vulnerable to adverse consequences of hospitalisation, including delirium, functional decline and hospital-acquired infections [4–6]. Furthermore, ~70% of care home residents have dementia and the hospital environment can be disorienting [7, 8]. Yet, the default response for older adults who experience deterioration is hospital admission [9]. In England, health policies emphasise reducing avoidable hospital admissions, delivering services in community and residential settings (including care homes) and improving integrated care, reflected in National Health Service (NHS) strategic plans to shift from hospital-based care towards community-based provision [10–12].

Hospital at Home (HaH) is an alternative model of care to hospitalisation. HaH offers hospital-level care in a person’s usual residence, providing monitoring, diagnostics and treatment that would otherwise require hospital admission [13]. It is designed to either avoid hospital admission or provide early supported discharge from hospital, which shortens the length of hospital stay [13]. Although well-established in the United States, France, Canada, Spain, the Netherlands and parts of the UK [14, 15], HaH services in the UK, have recently gained increased investment and focus [11, 12]. Sometimes referred to as ‘virtual wards’, the term HaH is advocated over virtual wards to ensure patients understand that care is provided primarily face-to-face, but can be supported by remote monitoring technology where appropriate [16].

Evidence suggests HaH can improve patient experience and outcomes, reduce hospital admissions and support health system efficiency [17]. However, little is known about how HaH services are delivered and experienced within care homes [18]. Care home staff are responsible for recognising early signs of deterioration, escalating concerns to healthcare providers, managing deteriorations and working with external healthcare teams to support residents [19]. Their perspectives are critical, both in understanding how HaH is experienced in care homes, and in identifying the challenges and opportunities it creates within the care home environment. Hence, this study aimed to explore care home staff’s experiences of HaH services while supporting physically unwell residents.

## Methods

### Study design

This paper reports findings from the qualitatively driven (QUAL-quan) component of a broader mixed-methods study [20] exploring care home staff experiences of health service utilisation and integrated care for unwell residents. While the overall study included both survey and interview data, the present analysis prioritises qualitative findings, with descriptive survey results used to support interpretation where appropriate.

Participants were care home staff recruited across six geographical regions in England, including Oxfordshire, Buckinghamshire, North-East Hampshire, Berkshire, Farnham and Surrey Heath.

Ethical approval for the study was obtained from the University of Oxford Research Ethics Committee (reference R90466/RE001).

### Sampling, recruitment and consent

Survey and interview tools were developed by the research team and refined with input from five care home staff (two nursing homes and three residential homes). Eligible participants were care home staff (managers, nurses, carers) aged ≥18 years, able to complete the survey and/or an interview in English. The survey was distributed to ~450 care homes across the six regions via the National Institute for Health and Care Research (NIHR) Enabling Research in Care Homes network, Immedicare (a regional telemedicine service), local care associations, professional networks and direct invitations via post or email.

An information sheet was provided at the survey entry point and consent was obtained electronically prior to access. The survey was open March–December 2024, with two reminder mailings.

Interviewees were recruited through survey opt-in and researcher networks. Of the 27 expressions of interest, 20 interviews were completed (February and December 2024); non-participation was due to loss of contact (n = 5), invalid details (n = 1), or workload (n = 1). Written consent was obtained prior to interviews taking place.

### Data collection

Audio-recorded interviews were conducted by telephone or Microsoft Teams, and guided by an interview schedule covering common reasons for physical deterioration, experience of HaH including benefits and challenges, preferred treatment models and experiences of residents’ hospitalisation.

The online survey delivered through Jisc (an online survey tool), was piloted with four researchers and one public contributor. The survey assessed the frequency of hospitalisation, admission avoidance HaH and early discharge HaH for care home residents. The survey sought to understand care home staff’s preferred treatment model for their residents. The survey was anonymous unless participants provided contact details for follow-up interviews.

### Data analysis

Quantitative survey data were analysed descriptively using Excel. Open-ended survey responses and interview transcripts were analysed thematically following the Braun and Clarke approach [21] using NVivo 15. Interviews were transcribed verbatim and anonymised prior to analysis. CN coded the transcripts. Three transcripts (15% of the dataset) were independently coded by MP and SM, with discrepancies discussed to refine the coding framework. Once established, the coding framework was applied to all transcripts with themes iteratively refined and illustrated using representative extracts. Descriptive survey findings are used to support interpretation of the interview data where relevant.

## Results

### Participants

Participant characteristics are summarised in Table 1.

**Table 1:** Participant characteristics

Characteristic	Survey (n = 50)	Interviews (n = 20)
**Role**		
Manager / Deputy Manager	25	14
Nurse / Nursing Associate	8	2
Healthcare Assistant / Care Team Lead	13	4
Ancillary Staff	4	0
**Setting**		
Residential / Supported Living	22	6
Nursing	20	11
Dual-registered	8	3
**Hospital at Home experience**	Not directly assessed	15
**Geographical location**		
Oxfordshire	26	11
Buckinghamshire	16	5
North-East Hampshire	4	0
Berkshire	3	3
Farnham	1	1
Surrey Heath	0	0

### Residents’ health needs and deterioration

Participants described residents experiencing both acute, sudden-onset conditions (e.g. fractures, infections, heart failure) and chronic or progressively worsening conditions (e.g. frailty), underscoring their complexity and vulnerability (Table 2). Recognising deterioration was central to determining the appropriate response. While some acute conditions required hospital admission (e.g. fractures), others (e.g. heart failure) could be managed within the care home with HaH support.

**Table 2:** Types of physical deterioration reported by care home staff

Type of deterioration	Common clinical problems where care home staff sought medical advice	Sudden or severe clinical problems that care home staff felt needed acute medical attention
Cognitive / Neurological	Cognitive impairment, dementia, Parkinson’s disease	Severe dementia, stroke with dysphasia, acute delirium
Physical / Mobility	Arthritis, recurrent falls without fracture	Falls with fracture, sudden immobility
Infection	Urinary Tract Infection, viral respiratory illnesses	Chest infection, cellulitis, sepsis
Skin / Tissue Integrity	Dry or fragile skin, early-stage pressure ulcer (Stage I–II)	Advanced leg or pressure ulcer (Stage III–IV), widespread or infected leg or pressure ulcer
Mental Health	Mild anxiety, depression, anorexia of ageing	Severe mental illness
Cardiovascular / Metabolic	Hypertension, diabetes, stable angina	Heart failure, acute kidney failure, unstable angina
Cancer / Chronic Disease	Early-stage COPD, early or controlled cancer	End-stage COPD, metastatic or rapidly progressing cancer
Continence	Occasional or frequent incontinence	Sudden or complete loss of continence
Frailty / Geriatric Syndromes	Pre-frailty, frailty (gradual decline, multiple deficits)	Severe frailty with dependency, multi-morbidity, or acute decompensation

Abbreviation: COPD, chronic obstructive pulmonary disease

### Findings

Three main themes were identified: (1) Experiences and consequences of hospitalisation, (2) Organisation and experiences of HaH and (3) Issues for implementation of HaH in care homes. These themes are presented below with representative quotes. Interview excerpts are identified by participant number (e.g. P1, P2), professional role and whether the participant had direct experience of HaH. Survey findings are incorporated within the themes where relevant (see Appendix S1 for illustrative quotes).

### Experiences and consequences of hospitalisation

Participants agreed that hospital admission was appropriate in some cases and should be assessed on an individual basis, particularly where diagnostic tests (e.g. X-rays for suspected fractures) or specialist treatment were required.

*Of course, without question, if anybody fractures anything, they would go straight into hospital … broken bones … they’ve come out and they’ve been good [P10*, *Manager*; *HaH experience]*

However, they noted that hospital staff were unfamiliar with residents’ usual behaviours and communication needs, especially for those with speech, language, or cognitive impairments. This lack of familiarity made care challenging and often distressing for residents.

*They have no idea who this person is … That is the biggest disadvantage … we have people here that have aphasia, for example. And they keep saying ‘oh I need to go to the train station’ … and what they actually mean is ‘I need to go to the loo’ [P6, Manager; HaH experience]*

Participants described residents removing intravenous (IV) lines, communication and information-gathering difficulties and agitation or aggressive behaviour linked to confusion. Hospitalisation was also associated with loss of comfort and independence, disruption to routine and reluctance to attend hospital.

#### Hospital delays and emotional distress

Participants reported that residents admitted to hospital frequently experienced delays, including long waits to be reviewed or allocated a bed. Prolonged hospital stays and delayed discharges—sometimes lasting from days to months—were also described. Although the specific causes were not always clear, some participants linked delays to staff shortages, limited bed availability, medication procurement issues and diagnostic backlogs. These experiences caused disappointment and emotional strain for residents and their families.

*A [resident’s] just been diagnosed with bile duct cancer … they went off to hospital … in the afternoon. And they didn’t come back until the following morning … She wasn’t admitted, she was waiting in her chair all night with her daughter … They’re the situations you really want to avoid for your [older and frail] residents [P11, Manager; HaH experience]*

Care home staff described efforts to minimise distress by ensuring residents were accompanied by a family member or staff member where possible; however, this was not always feasible. Particular concern was expressed for residents living with dementia, who may not recognise their surroundings, understand what was happening, or attend to basic needs such as eating and drinking.

These views were echoed in open-ended survey responses, where respondents indicated that residents generally preferred treatment within the care home and viewed hospital admission as unnecessary in most cases unless a fracture had occurred.

#### Breakdowns in communication and continuity

Participants reported significant communication challenges during hospital admissions, including residents’ information being lost or overlooked and care home staff having to repeatedly provide the same details. Concerns were also raised about medication management and discharge processes. They described difficulties obtaining timely updates, with some residents returning to the care home without prior notice or a discharge letter. These breakdowns were a major source of frustration and tension between hospital and care home staff.

*Communication is the biggest, biggest issue … we’re just not kept in the loop … We’re not always contacted if they’ve been discharged … we’re not always given a discharge letter, so we don’t know if medications have been changed [P20, Deputy manager; HaH experience]*

Instances of premature discharge, particularly during weekends and bank holidays, which sometimes led to deterioration and re-admission, were also reported.

*[A resident] … had surgery [for a broken hip], and they called us within four days and said she’s ready to come home … We’re a residential home … we actually went up and [assessed her] … she wasn’t eating and drinking… couldn’t mobilise … had delirium* … *If she’d have come back … it would have been a failed discharge [P14, Manager; HaH experience]*

Survey respondents similarly described poor interprofessional communication and siloed working across services, as well as liability concerns for care homes when residents were discharged prematurely, without medication, or appropriate preparation.

#### Hospital-associated decline and deterioration

Participants identified several forms of hospital-associated deterioration among care home residents (Table 3), including functional decline, hospital-acquired infections, and cognitive deterioration. Hospitals were described as primarily focused on acute and emergency care, and this—alongside staffing shortages—was perceived to contribute to residents’ decline. Participants raised concerns about the suitability of hospital environments, characterising them as overwhelming, impersonal and detrimental to residents’ wellbeing.

**Table 3:** Types and outcomes of hospital-associated deterioration

Type of deterioration	Key features	Outcome	Example quote
Hospital-associated functional decline	Loss of mobility, onset or worsening of incontinence	Physical deterioration, reduced independence, changes to care package requirements	*We often find that the residents are very much bedbound when they’re in hospital . . . if we can keep the resident here, we will do our utmost to keep them here because obviously the care they get in hospital can be quite challenging because we know they have staffing issues [P3, Manager; HaH experience]* *One of our older clients . . . stopped walking, because she’d been in hospital for two months . . . she was put in pads and just left to be incontinent . . . That then really changed her needs, and when she came home her care package had to change [P13, Deputy Manager; No HaH experience]*
Hospital-associated adverse outcomes	Pressure sores, dehydration, sore mouth and depression	Physical and emotional/cognitive deterioration	*Our patients being in hospital, we find really, really upsetting because they, more often than not, come back with pressure sores, dehydration, sore mouths, unclean, left in hospital gowns, left uncared for, unkempt and very, very, very, very depressed and upset . . . so are the families [P16, Manager; HaH experience]*
	Weight loss and malnutrition	Nutritional decline and marked reduction in overall wellbeing	*We know there’s challenges of residents being assisted to eat and drink in the hospital, very often . . . they come back with quite significant weight loss [P3, Manager; HaH experience]*
Hospital-acquired infection	Pneumonia, *E. coli*, Norovirus, COVID-19	Iatrogenic decline, further hospitalisation and increased vulnerability	*Many of our residents that end up going into hospital, nine times out of ten, they end up contracting something else from the hospital … a resident recently [went] in due to a leg injury and she’s now got pneumonia and E.coli since being in hospital. This can happen quite frequently . . . norovirus or . . . COVID . . . it’s being with a lot of other people that are obviously really sick in the hospital [P20, Deputy Manager; HaH experience]*
Hospital-associated cognitive decline or delirium	Acute confusion, exacerbation of dementia, disorientation	Deterioration in mental state and psychological decline	*We don’t really like sending people into hospital because they come back even more confused, their dementia tends to aggravate because it’s a new environment. They’re very disorientated, they don’t know them [hospital staff] so it creates a lot of problems [P6, Manager; HaH experience]*

*I think we should avoid hospital admissions at all costs, when it comes to older people … they’re busy, it’s loud, you don’t really have any privacy because you’re in a bay. It’s a lot of people that they don’t know … they should be looked after here, definitely, not sent to hospital [P6, Manager; HaH experience]*

Some residents admitted for acute illness did not recover, and in some cases, died away from home. Those who returned frequently required increased support with personal care, toileting, hydration, mobility and other activities of daily living. Survey respondents attributed this hospital-associated functional decline to limited holistic care during hospital admission and viewed familiar surroundings as more supportive of recovery.

Survey data further indicated that most respondents (n = 34, 69%) favoured avoiding hospital admission through admission avoidance HaH (n = 23, 47%) or early discharge HaH (n = 11, 22%), while a smaller proportion (n = 12, 24%) preferred residents to remain in hospital until fully recovered. However, hospital admission followed by return to the care home remained the most frequently reported care pathway (Table 4), with admission avoidance and early discharge HaH used less often.

**Table 4:** Types of hospital-related care received by residents

	Frequency N(%)		
Treatment model	Always /often	Sometimes	Rarely/never
Admitted to hospital and returns to the care home when recovered	34 (68)	12 (24)	2 (4)
After discharge continues hospital treatment or care in the care home (early discharge HaH)	20 (42)	21 (44)	5 (10)
Receives hospital treatment or care in the care home (admission avoidance HaH)	19 (39)	20 (41)	6 (12)

Together, the qualitative and survey findings indicate a clear preference among staff for HaH, despite hospital remaining the dominant care pathway in practice.

### Organisation and experiences of HaH

Participants described considerable variation in the organisation and delivery of HaH services across areas. They also reflected on their experiences of the model, highlighting both its perceived benefits for residents and the practical challenges associated with its implementation.

#### Organisation and delivery of HaH

The organisation of HaH services varied across areas. In some localities, services were delivered through GP practices or hospital-based teams (e.g. rapid access care units or respiratory services), while others were managed from community hubs. Referral arrangements also differed; in some areas, GPs acted as gatekeepers, whereas in others, care homes could refer directly.

*For the Hospital at Home, it needs to be through the GP so we cannot refer them directly. It’s the only service … that the GP is the one that organises them to come to us [P9, Manager; HaH experience]*

Participants suggested that HaH expanded during the COVID-19 pandemic, though the length of time services had operated in individual homes was unclear. Access varied widely, from a small number of residents to most or all residents. Communication was typically by phone, and most staff used their own equipment, although some services provided digital tools for monitoring.

*We have to ring the respiratory team and onboard the resident and then … do two sets of observations a day which are inputted into the respiratory team’s tablet, which we’ve got onsite. It then is automatically reviewed by the respiratory team at the hospital [P3, Manager; HaH experience]*

Care home staff reported receiving varied training to support residents, though this was not always specific to or delivered by the HaH service. Nursing home staff generally felt confident supporting HaH, whereas residential staff described receiving task-focused guidance, particularly around monitoring IV lines and recognising deterioration, with instructions to escalate if concerns arose.

HaH provision ranged from end-of-life care to acute interventions such as IV therapies, diuretics, blood transfusions and point-of-care diagnostics to support clinical decision-making and avoid hospital transfer. Visit frequency was typically once or twice daily, treatment duration ranged from a few days to several weeks, and some treatments, such as management of heart failure or fluid overload, required longer involvement. Situations warranting their attention included recurrent infections, kidney problems, end-of-life/palliative care, (advanced) heart failure, fluid overload, COPD and sepsis. Between visits, care home staff resumed responsibility for monitoring and escalation.

*[A resident] … had fluid overload because of his heart failure, and he was on maximum dose of oral bumetanide, but he required the IV dose … The district nurse didn’t have the capacity. It then fell back to hospital at home to do that treatment … [for] as long as it’s required [P14, Manager, HaH experience]*

#### Benefits of HaH

Participants described multiple benefits of HaH at the resident, professional and system levels. These are described in turn below:

##### Resident-level benefits

Nearly all participants viewed HaH positively, considering treatment within the care home as more appropriate for older and frail residents. Avoiding hospital was seen to reduce disruption, delays and associated emotional and physical burden.

*The average age of our residents here is 90, so can you imagine residents queuing at the A&E? Just to get an antibiotic … that could just [have] been given here [P12, Manager; HaH experience]*

Participants expressed concern that residents were often unable to advocate for themselves during hospital treatment, increasing their vulnerability and the likelihood that their needs would be overlooked or misunderstood. HaH was viewed as enabling more individualised and responsive care, with care home staff present to recognise subtle changes and communicate residents’ preferences and distress. Remaining in familiar surroundings, supported by trusted staff, was seen to promote dignity and comfort—particularly at end-of-life—while continuity of care contributed to a more peaceful, person-centred experience.

*If the patient is dying, then they have a good death … If they recover, they tend to recover quicker and better … knowing and understanding their environment, even if they’ve got dementia [P16, Manager; HaH experience]*

Most participants without prior HaH experience similarly viewed it as a valuable model and expressed a desire for hospital-level treatments to be delivered within care homes. Receiving prompt and appropriate care within a familiar environment was perceived to reduce exposure to hospital-associated risks. Some participants also believed HaH contributed to improved health outcomes and longevity.

##### Professional benefits

Participants described strong collaboration between the HaH team and care home staff, characterised by mutual respect, and shared responsibility for residents’ recovery. This partnership was viewed as central to reducing hospital admissions.

*Hospital at Home … are amazing … They come; they speak with us … they advise us ... they know they need to work with us for the recovery of the patient to avoid hospitalisation [P9, Manager; HaH experience]*

HaH services were perceived to strengthen relationships between hospitals and care homes and to support staff development through training and shadowing opportunities.

Participants expressed strong confidence in the HaH team’s expertise and compassionate approach. They described staff as efficient, knowledgeable and highly skilled, while also demonstrating empathy and kindness in their interactions.

*They’re very efficient … they know their stuff and they provide a lot of compassion … to the relatives, especially when … residents are on end-of-life … It’s providing that support and guidance for the staff as well [P20, Deputy manager; HaH experience]*

Participants valued the continuity and coordination provided by HaH services, which they perceived as facilitating smoother transitions from hospital to the care home and improving communication between services.

*They will pick the person up in A&E and then follow their journey when they come back to the home. They quite often call us from A&E and [say] … I just want to let you know that we’ve picked [name] up and when he’s discharged, we’ll come and see him at the home. That’s been very helpful [P2, Manager, HaH experience]*

##### System-level benefits

Participants described HaH as a progressive model that aligned with the future direction of healthcare delivery. It was viewed as beneficial for the wider health system due to alleviating pressure on hospitals and making more efficient use of resources.

*I personally think it’s the way forward. It’s going to free up hospital beds … if their care can be carried out in their care home, that’s going to take a lot of pressure off the hospitals … Most of the residents … don’t want to go into [or] die in hospital [P11, Manager; HaH experience]*

Survey respondents similarly described HaH as a sustainable alternative to hospital admission, however, current provision was noted to be limited.

#### Limitations or challenges of HaH

Participants identified several challenges that limited the reach and effectiveness of HaH in certain situations.

##### Managing transmission of infectious diseases within the care  home

One healthcare assistant without prior HaH experience questioned the benefit of treating infections in the care home. They suggested that such cases may be better managed in hospital settings where infection control measures could be more effectively implemented, thereby reducing the risk of transmission among vulnerable residents and staff.

##### Constrained access to HaH services

Participants identified capacity constraints as a significant limitation of HaH. Referrals were sometimes declined due to lack of availability, resulting in delays and, in some cases, emergency hospital transfer.

*I do not think the [HaH] system is robust enough … I had situations where we thought Hospital at Home was coming but no one showed … we chased up with the GP, and the GP says they don’t have any spaces, you’re going to need to call 999 ... you call 999 … then you wait another six hours [P9, Manager; HaH experience]*

Limited out-of-hours provision was also highlighted. While the service was described as responsive during the day, its absence overnight created gaps in continuity and access to urgent care.

*We find that they come in … usually eight o'clock in the morning until eight o'clock at night … People think hospital at home means they’re going to be there twenty-four-seven, they’re not [P16, Manager; HaH experience]*

Some participants described difficulty accessing HaH due to reliance on GP referrals, which was perceived as a barrier to timely support particularly when GPs were unavailable or unwilling to make a referral. Care home staff expressed frustration that their ability to secure HaH support for residents was limited by these procedural constraints.

*We can’t refer to hospital at home. So, the GP has to do that. So, it all, kind of, lies on whether the GP will do that or not … the last time when we had [HaH], one of the families went to the [practice], and … had to beg the doctor to get the hospital at home. He reluctantly did it … But it was difficult to get. Very difficult [P17, Healthcare assistant/team lead; HaH experience]*

##### System level gaps in continuity and safety

Participants expressed concern about the lack of coordination between hospital discharge teams and the HaH service. They described situations where residents were discharged prematurely without post-discharge support from HaH, leaving care home staff to manage residents whose needs remained complex or unstable.

*They will discharge early but they won’t arrange Hospital at Home … Sometimes they will discharge some persons even though they have the same symptoms that they had before the admission [P7, Registered nurse; HaH experience]*

These perceived systemic gaps between hospital and community care often resulted in a ‘boomerang’ effect of repeated hospital admissions. Participants believed that inadequate follow-up and reliance solely on GP care left residents without sufficient medical support to recover, leading to deterioration.

*We’ve had a lot of early discharges that ended up being like a boomerang … they send them back and we have no choice but to send them back into hospital … they don’t discharge them with [HaH], they just discharge them back to the care of the GP … the absence of … the extra support … will just crash them [P6, Manager; HaH experience]*

##### Family perceptions and acceptance of HaH

Some noted that relatives’ attitudes could pose challenges to HaH utilisation. Certain family members were described as anxious or reluctant to accept it, perceiving hospital admission as the safest or most appropriate option for their loved one.

*I would think the majority [of family/friends] would be very much in favour, there are obviously some relatives who are very anxious who hospital is hospital all the way because of their own anxieties and lack of understanding potentially [P3, Manager; HaH experience]*

### Issues with implementation of HaH in care homes

Some participants emphasised that successful expansion of HaH would require targeted training to support care home staff. Nursing home staff suggested that their nurses could contribute by being trained to deliver hospital-level interventions within care homes. Residential homes similarly highlighted the importance of improving staff proficiency in recognising deterioration and escalating concerns. Overall, participants indicated that wider implementation would depend on sufficient workforce capacity, geographical coverage and appropriately skilled personnel.

*If they weren’t a big enough team and they had to travel quite a distance … that could delay treatment for a resident … It’s having staff who are confident enough to deal with the situation … and it’s the clinical team being able to get to the care home quickly [P11, Manager; HaH experience]*

Improving workforce capacity was viewed as essential to ensuring timely access and maximising HaH’s potential to reduce avoidable hospital admissions.

*If we had more capacity of the Hospital at Home services, we would avoid further admissions in hospital [P9, Manager, HaH experience]*

Participants also emphasised the need for greater investment and expansion to make the service more robust and accessible to care homes. Without adequate resources and scale, availability and response times were perceived to limit its overall impact.

*It needs to be a bigger service … for the elderly and the frail, the Hospital at Home service needs to be really looked into, expanded, funded, because it’s an excellent service for that cohort of people [P2, Manager; HaH experience]*

Participants emphasised the importance of effective communication and continuity during hospital transfers, including timely updates, clear documentation and stronger collaboration between hospital, HaH and care home teams.

*I really like the idea of the joined-up working … bridging that gap between hospital and us … [HaH] would definitely help with … joined up working between the teams … partnership working … would be great [P13, Deputy manager; No HaH experience]*

Effective coordination across services was viewed as critical to the success of early discharge. Participants highlighted the need to balance discharge timing carefully, noting that both premature and delayed discharge could result in deterioration and re-admission.

## Discussion

This study is, to the best of our knowledge, the first to explore the perceptions and experiences of care home staff about HaH services. Findings suggest a clear tension between the default model—hospitalisation and the new model—HaH. Hospitalisation is still more common but care home staff strongly believe that many residents would fare better if treated within the care home environment via HaH. Participants widely endorsed the principle of HaH as a more person-centred, dignified and safer alternative, and highlighted several negative consequences of hospitalisation for care home residents, including functional decline, deconditioning, hospital-acquired infections and cognitive deterioration, echoing previous literature describing hospital admission as high-risk for frail older adults [22, 23].

Participants noted that some residents never returned to the care home after hospitalisation and those who did often required more intensive support with mobility, hydration and personal care. Existing research has shown that even short hospital admissions can lead to loss of independence, poorer quality of life and mortality [23–25]. Hospital environments were described as unfamiliar and overstimulating, and particularly distressing for residents with dementia. This aligns with evidence that disruption of routine and environment exacerbates confusion and behavioural symptoms among cognitively impaired adults [26].

Participants also raised concerns about communication and coordination across settings. Information loss, premature or delayed discharge, missing discharge summaries, medication changes and unannounced resident returns were cited as major contributors to avoidable readmissions in line with other literature [27, 28]. Staff attributed many challenges to systemic hospital pressures, particularly bed demand and staffing shortages. While empathising with these constraints, they stressed the need to balance efficiency with safe, person-centred discharge planning. HaH was viewed as a progressive alternative that enabled hospital-level care in familiar surroundings while reducing pressure on hospital beds. These perceptions resonate with evaluations of HaH services demonstrating equivalent or improved clinical outcomes compared with inpatient care, alongside higher patient satisfaction and reduced costs [17]. Staff valued HaH’s emphasis on partnership, collaboration, responsive communication and integrated care—aligning with recent evidence and policy calls for greater coordination between acute and community services to reduce avoidable admissions among older adults [12, 29].

Importantly, participants highlighted the relational advantages of providing care within familiar environments. In the care home, staff knew residents’ personalities, routines and ways of communicating, allowing them to recognise subtle changes in condition or distress. This familiarity compensated for residents’ reduced ability to advocate for themselves, particularly those with dementia or speech and language difficulties, consistent with person-centred care frameworks [30]. The findings align with a growing body of research advocating for community-based alternatives to hospital care for older adults [17, 31–34]. The continuing dominance of hospital-based model in the quantitative data underscores the challenge of embedding HaH models within care homes. Staff believed that hospital admission was not always in residents’ best interests or in line with their wishes, reinforcing policy calls for advanced care planning and shared decision-making to ensure treatment aligns with residents’ preferences [35].

The survey results offer a useful contrast to the qualitative narratives. The finding that 68% of respondents reported hospital admission (with return to the care home post-recovery) as occurring always or often—compared with 39% for admission-avoidance HaH and 42% for early-discharge HaH—underscores a central issue. Although the benefits of HaH for older adults are widely recognised by participants and reflected in policy, delivery in practice remains constrained, highlighting the tension between policy aspiration and operational reality. This aligns with wider evidence [36–40] that implementation of HaH services is limited by workforce and resource constraints, restrictive referral processes and uneven regional provision.

### Recommendations for policy and practice

1. **Integrated health and social care services for care home residents**

**Recognise and support care home staff expertise** by valuing their knowledge of residents and providing appropriate clinical support during episodes of acute deterioration.
**Develop tailored community care pathways** for acutely unwell care home residents, including clear triage processes, and improved access to primary and community services.
**Strengthen communication and digital integration** across health and social care through shared electronic records, accessible advance care plans, timely medication updates and improved information transfer during transitions between services.
**Improve hospital care for care home residents** by tailoring services to their needs, supporting rehabilitation and reducing iatrogenic harm through better coordination with care homes.

1. **Integrated Hospital at Home pathways for care home residents**

**Expand equitable access to HaH** for care home residents through sustained investment, increased workforce capacity and geographical coverage, direct referral routes, extended out-of-hours provision and alignment of services with the needs of residents and care home staff.
**Support and train care home staff to work with HaH services,** recognising differences between nursing and residential homes, and providing training to strengthen skills in monitoring and escalating physical deterioration.
**Establish direct access routes for care homes** to raise concerns and obtain timely medical support for HaH patients, and ensure access to appropriate equipment for monitoring, documentation and medication administration.

### Strengths and limitations

A key strength of this study is the use of multiple data sources, combining qualitative interviews with descriptive survey data to provide a nuanced understanding of care home staff experiences and perceptions. Inclusion of participants from a range of care home settings further enhanced the diversity of perspectives captured.

The survey achieved a modest response rate (n = 50). This reflects well-documented challenges associated with conducting research in care home settings. These include significant workload pressures, limited staff capacity and time, communication barriers and variable levels of research awareness and engagement [41–44].

To mitigate these challenges, an extensive multi-pronged recruitment strategy was adopted, incorporating varied recruitment channels, incentives and an extended recruitment period (≥9 months) [41]. Despite this, overall participation remained limited, highlighting persistent structural and organisational barriers to care home research. It is also possible that respondents were those with greater interest in research, which should be considered when interpreting the findings.

The modest survey sample limited the scope for quantitative analysis and precluded meaningful comparisons across professional groups and regions. It is also possible that differences in service provision between regions are underrepresented, particularly in areas with very small or no recruitment. Experiences from less represented areas may therefore be less visible in the data. However, in-depth interviews, supported by open-ended survey responses, provided rich insight into staff views and captured a range of perspectives.

Finally, the findings are limited to care home staff and do not include hospital staff or residents’ perspectives, which may restrict understanding of inter-organisational constraints, discharge decision-making and residents’ experiences.

## Conclusion

This study highlights a clear gap between policy ambition and practice: although care home staff advocate for delivering hospital-level care within care homes, hospitalisation often remains the default pathway. Participants viewed HaH as a valuable and safer alternative that preserves continuity, reduces distress and supports recovery. However, limitations in service capacity, referral pathways, operating hours and geographical coverage restrict wider implementation. Addressing these constraints will require sustained investment, improved coordination between hospital, HaH and care home services, and a focus on delivering care in settings that best support safety and quality of life. Further research is needed to explore residents’ experiences and perspectives.

## Supplementary Material

Supplementary_materials_afag236

## Data Availability

The data that support the findings of this study are not publicly available due to ethical and privacy considerations, as they contain information that could compromise the confidentiality of participants. Anonymised data may be available from the corresponding author on reasonable request, subject to appropriate ethical approvals.
