# Supplementary material for: Hospital at Home for older residents with physical deterioration: care home staff experiences
Source: Age Ageing. 2026 Aug 11;55(8):afag236. doi: 10.1093/ageing/afag236 (PMC13461218; doi:10.1093/ageing/afag236)
Supplement: Supplementary file 1 [file supplementary_materials_afag236.docx]

**Hospital at Home for older residents with physical deterioration: Care home staff experiences**

**Appendix 1**	**Open-ended survey responses on hospitalisation and HaH**

| **Subtheme** | **Key Insight** | **Illustrative Quotes** |
| --- | --- | --- |
| 1. Hospital-associated decline and deterioration | Hospitalisation was widely perceived as harmful for frail older residents, leading to functional decline, distress, and poor recovery. Participants felt hospitals focus on treatment rather than holistic care. | *Most do not require hospital treatment and hospital admission is detrimental to health [Manager]*  *In general, residents are better with us than hospital. They come back malnourished, dehydrated, often bruised, have lost mobility and sometimes never recover use of legs. In my opinion they receive treatment but not care [Health care assistant]*  *Unless a resident has a broken bone then hospital admission is futile … even a couple of hours in A&E can do significant damage to health and wellbeing [Manager]* |
| 2. System-level gaps in continuity | Participants reported concerns about premature discharge, lack of follow-up, and poor communication between hospitals and care homes, leading to avoidable readmissions. | *When admitted to hospital residents are sent home without treatment or follow up until homes chase up updates with GP, this happens mainly during weekend/bank holidays [Manager]*  *Very often discharged prematurely and the deterioration continues [Registered nurse]* |
| 3. Preference for care in familiar environments | Staff felt residents recovered better when treated in their own care homes, where they were surrounded by familiar people and routines. Remaining in place was viewed as promoting comfort, dignity, and better health outcomes. | *Avoiding hospital admission and avoiding more distress and health deterioration in hospital is the best decision [Deputy manager]*  *Elderly people who become physically unwell should be given care where they are if at all possible as it has been shown they make better recovery [Registered nurse]*  *Care for care home residents in the care home and have open discussions about end of life and when medical treatment is not in the residents’ best interests [Manager]* |
| 4. Benefits of Hospital at Home (HaH) services | Participants endorsed expansion of HaH, viewing this as a progressive and person-centred alternative that prevent unnecessary hospital admissions and improve resident wellbeing. | *There is a hospital at home team, frailty team; however, the service is small. When we have had them attend a resident in the home this has prevented hospital admissions [Manager]*  *It would be great if a service could be offered … allowing IV antibiotics to be administered by community clinicians within the care home. This would avoid some hospital admissions, free up hospital beds and would have a positive effect on the resident’s wellbeing [Manager]* |
| 5. Balancing admission and discharge decisions | Participants highlighted the importance of assessing residents individually and ensuring hospital discharge only when treatment is complete to prevent deterioration and re-hospitalisation. | *The best for the residents is to stay at home as majority of them prefer this … if they are admitted, they should only be sent back once treatment is complete, as often this is not happening and they are soon readmitted [Manager]* |
